# Glucagon-Like Peptide-1 Receptor Agonist Order Fills and Out-of-Pocket Costs by Race, Ethnicity, and Indication

**DOI:** 10.1001/jamahealthforum.2025.4258

**Published:** 2025-10-10

**Authors:** Ameet Sarpatwari, Mark J. Soto, Ishani Ganguli, Caroline E. Sloan, Foster Goss, Anna D. Sinaiko

**Affiliations:** 1 Division of Health Policy and Insurance Research, Department of Population Medicine, Harvard Pilgrim Health Care Institute and Harvard Medical School, Boston, Massachusetts; 2 Department of Health Policy and Management, Harvard T.H. Chan School of Public Health, Boston, Massachusetts; 3 Division of General Internal Medicine and Primary Care, Department of Medicine, Brigham and Women’s Hospital and Harvard Medical School, Boston, Massachusetts; 4 Department of Medicine, Duke University, Durham, North Carolina; 5 University of Colorado Hospital, Aurora; 6 University of Colorado School of Medicine, Aurora

## Abstract

This cohort study examines how often glucagon-like peptide-1 receptor agonist orders are filled, the out-of-pocket cost per prescription, and differences by patient race and ethnicity or indication among patients with insurance.

## Introduction

Prescribing of glucagon-like peptide-1 receptor agonists (GLP-1RAs) for diabetes and obesity has soared, yet patients report barriers accessing these medications.^1^ The impact of out-of-pocket (OOP) costs on access is concerning for patients in racial and ethnic minority groups, who have higher prevalence of these conditions, and can face racial disparities in GLP-1RA receipt,^1,2,3^ and for patients with obesity alone, who can be excluded from insurance coverage.^4^ The 2024 average retail price of GLP-1RAs—paid by those without insurance—was more than $900 per month.^5^ Among patients with insurance, how often GLP-1RA orders are filled, the OOP cost per prescription, and differences by patient race and ethnicity or indication are unknown.

## Methods

In this cohort study, we linked medication orders and patient characteristics from the University of Colorado Health system (UCHealth) electronic health record (EHR) to eligibility, medical, and pharmacy claims from the Colorado All-Payer Claims Database. We included patients with 1 or more of 15 common chronic conditions who had a GLP-1RA order between January 2018 and September 2022 and continuous enrollment in Medicare or commercial insurance for 12 months prior and 3 months after the order (eFigure 1, eTable 1 in Supplement 1). We measured whether the order was filled within 90 days, and for filled orders, the patient’s OOP cost per 30-day supply (inflation-adjusted to 2023 dollars). We estimated differences in outcomes by patient race and ethnicity (Hispanic, non-Hispanic Asian, non-Hispanic Black, and non-Hispanic White) and by drug indication (diabetes only, obesity only, diabetes and obesity, neither) using logistic and generalized linear regression controlling for age, sex, insurance type, geography, median household income by zip code, and order type (initial or refill), clustering standard errors at the patient level (eMethods in Supplement 1). We considered *P* < .05 to be significant. Data analysis took place from January to December 2024. This study followed the Strengthening the Reporting of Observational Studies in Epidemiology (STROBE) reporting guidelines^6^ and was approved by the Colorado Multiple and Harvard Longwood Campus institutional review boards, which waived informed consent.

## Results

**Figure 1.**
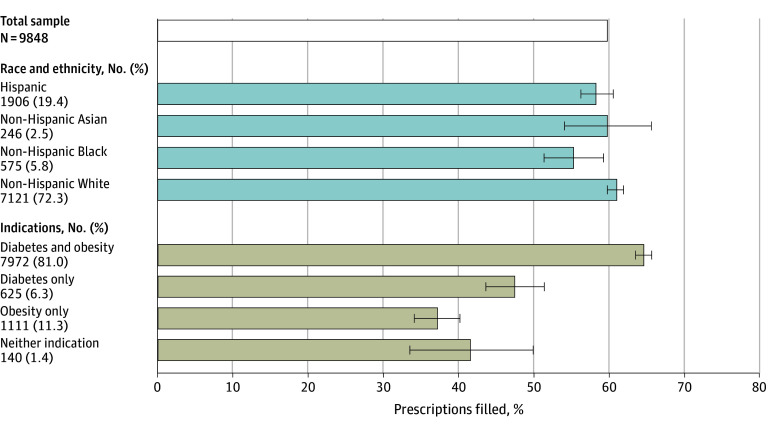
Rate of Fill of Glucagon-Like Peptide-1 (GLP-1) Receptor Agonist Orders by Race, Ethnicity, and Indication Sample includes GLP-1 medication orders from January 1, 2018, to September 30, 2022. The top bar reflects the fill rate for the sample. The bars below report predicted fill rates by each group adjusted for age, sex, insurance type (traditional Medicare, Medicare Advantage, commercial), urban or rural geography, median household income by zip code, and order type (initial or refill) using coefficients from logistic regression models with month and year fixed effects and standard errors clustered on individual patients. The whiskers indicate the 95% CIs. All categories have a reported value (ie, there is no reference group).

The sample included 9848 GLP-1RA orders for 6094 unique patients. The mean (SD) patient age was 60.9 (12.7) years; 5285 were women (53.7%); 246 were non-Hispanic Asian (2.5%), 575 were non-Hispanic Black (5.8%), 1906 were Hispanic (19.4%), and 7121 were non-Hispanic White (72.3%). Overall, 5915 (60.1%) of GLP-1RA orders were filled. Non-Hispanic Black and Hispanic patients were less likely to fill their orders than non-Hispanic White patients (fill rate, 55.3% and 58.4% vs 60.9%; *P* = .006 and *P* = .045, respectively). Patients with both diabetes and obesity were more likely to fill their order than were patients with diabetes only or obesity only (64.6% vs 47.5% vs 37.2%; *P* < .001) (Figure 1).

**Figure 2.**
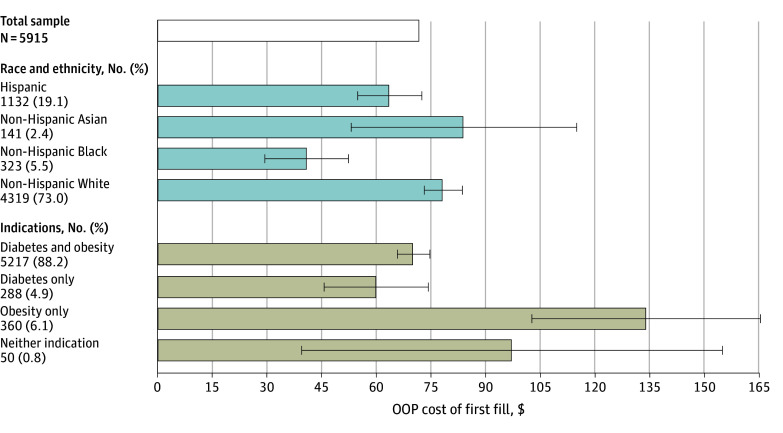
Out-of-Pocket (OOP) Cost per Prescription for a Glucagon-Like Peptide-1 (GLP-1) Receptor Agonist, 30-Day Supply The number of filled orders within each category (sample size) is reported under each category label on the y-axis. The top bar reflects the mean (median) OOP cost for a 30-day supply. The bars below report predicted OOP costs adjusted for age, sex, insurance type (traditional Medicare, Medicare Advantage, commercial), urban or rural geography, zip code median household income, and order type (initial or refill) using a multivariable generalized linear model log link function and γ distribution (see Supplement 1 for additional detail). Models included year and month fixed effects, and robust standard errors clustered on individual patients. The whiskers indicate the 95% CIs. All categories have a reported value (ie, there is no reference group).

Among filled GLP-1RA orders, mean (SD) OOP cost was $71.90 ($163.69). OOP costs per order for non-Hispanic Black and Hispanic patients were lower than for non-Hispanic White patients ($41.15 and $63.69 compared with $78.37; *P* < .001 and *P* = .006, respectively), and lower for patients with diabetes and obesity than for patients with obesity alone ($70.32 compared with $134.04; *P* < .001) (Figure 2).

## Discussion

In this cohort study, 40% of orders for GLP-1RAs were not filled. Non-Hispanic Black patients and Hispanic patients were less likely to fill orders than non-Hispanic White patients, and the non-Hispanic Black and Hispanic patients who filled their orders paid lower OOP costs. OOP differences across groups may stem from differences in insurance coverage, use of different GLP-1RAs, or different cost thresholds for forgoing GLP-1RAs. OOP costs for patients with obesity alone were nearly 2 times those for patients with diabetes, likely reflecting less comprehensive insurance coverage for this indication.

This cohort study had some limitations. We included only 1 health care system, and we could not assess reasons for nonadherence, or if medications were purchased using cash instead of insurance. The study period preceded widespread use of GLP-1RAs that are highly effective for obesity, and conclusions regarding results for obesity should be viewed with caution. Policymakers should explore options to improve equitable access to GLP-1RAs.

## Notes

Supplement 1. eMethods. eFigure 1. Study Sample Selection eTable 1. Chronic Conditions Included in Sample eTable 2. GLP-1 Receptor Agonist (GLP-1RA) Medications Included in Study

Supplement 2. Data Sharing Statement

## References

[1] Kim C, Ross JS, Jastreboff AM, . Uptake of and disparities in semaglutide and tirzepatide prescribing for obesity in the US. JAMA. 2025;333(24):2203-2206. doi:10.1001/jama.2025.4735 40299371 PMC12042086

[2] National Diabetes Statistics Report. Centers for Disease Control and Prevention. Published May 15, 2024. Accessed April 28, 2025. https://www.cdc.gov/diabetes/php/data-research/index.html.

[3] Petersen R, Pan L, Blanck HM. Racial and ethnic disparities in adult obesity in the United States: CDC’s tracking to inform state and local action. Prev Chronic Dis. 2019;16:E46. doi:10.5888/pcd16.180579 30974071 PMC6464044

[4] Medicare Coverage of Anti-Obesity Medications. ASPE Issue Brief. Published November 26, 2024. Accessed April 28, 2025. https://aspe.hhs.gov/sites/default/files/documents/127bd5b3347b34be31ac5c6b5ed30e6a/medicare-coverage-anti-obesity-meds.pdf.

[5] Williams E, Rudowitz R, Bell C. Medicaid Coverage of and Spending on GLP-1s. Kaiser Family Foundation. Published November 4, 2024. Accessed April 28, 2025. https://www.kff.org/medicaid/issue-brief/medicaid-coverage-of-and-spending-on-glp-1s/

[6] von Elm E, Altman DG, Egger M, Pocock SJ, Gøtzsche PC, Vandenbroucke JP; STROBE Initiative. The Strengthening the Reporting of Observational Studies in Epidemiology (STROBE) statement: guidelines for reporting observational studies. Lancet. 2007;370(9596):1453-1457. doi:10.1016/S0140-6736(07)61602-X 18064739

