# Supplementary material for: Glucagon-Like Peptide-1 Receptor Agonist Order Fills and Out-of-Pocket Costs by Race, Ethnicity, and Indication
Source: JAMA Health Forum. 2025 Oct 10;6(10):e254258. doi: 10.1001/jamahealthforum.2025.4258 (PMC12514621; doi:10.1001/jamahealthforum.2025.4258)
Supplement: Supplement 1. — eMethods. eFigure 1. Study Sample Selection eTable 1. Chronic Conditions Included in Sample eTable 2. GLP-1 Receptor Agonist (GLP-1RA) Medications Included in Study [file jamahealthforum-e254258-s001.pdf]

Supplemental Online Content

Sarpatwari A, Soto MJ, Ganguli I, et al. Glucagon-like peptide-1 receptor agonist order fills and out-of-pocket costs by race, ethnicity, and indication. *JAMA Health Forum*. 2025;6(10):e254258. doi:10.1001/jamahealthforum.2025.4258

eMethods.
eFigure 1. Study Sample Selection
eTable 1. Chronic Conditions Included in Sample
eTable 2. GLP-1 Receptor Agonist (GLP-1RA) Medications Included in Study

This supplemental material has been provided by the authors to give readers additional information about their work.

© 2025 Sarpatwari A et al. *JAMA Health Form*.

eMethods

This cohort study followed the Strengthening the Reporting of Observational Studies in Epidemiology (STROBE) reporting guideline. This study was approved by the Colorado Multiple and Harvard Longwood Campus Institutional Review Boards and informed consent was waived because we used deidentified data.

Study Setting: Our study was conducted at the University of Colorado Health (UCHealth), a large integrated community and academic health system that at the time of the study included 12 hospitals and over 600 ambulatory clinics. UCHealth uses the Epic® Electronic Health Record (EHR).

Data. From the University of Colorado Health System (UCHealth) (EHR) data warehouse, we selected patients with any one of 15 chronic conditions requiring medications that have been identified as highly prevalent among patients in the United States (**eTable 1**)[1,2] who received a medication order for a glucagon-like-peptide-1 receptor agonist (GLP-1RA) (**eTable 2**) between July 1, 2018 – September 30, 2022, and who had either Medicare or commercial insurance at the time of the order. Patients with Medicaid pay $0 for preferred medications; thus we excluded patients with Medicaid from this study. Patient race and ethnicity were reported directly in the UCHealth Electronic Medical Record data. We include this measure so that this study could assess whether there are difference in access and out-of-pocket spending on GLP-1RAs by race and ethnicity. Included individuals' identifiers were submitted directly from the UCHealth electronic health record data warehouse to the Center for Improving Value in Health Care (CIVHC), the organization that administers the Colorado All-Payer Claims Database (APCD).

© 2025 Sarpatwari A et al. *JAMA Health Form*.

The Colorado All-Payer Claims Database (APCD) includes monthly eligibility, medical claims, and pharmacy claims for 74% of insured individuals in Colorado. In 2022, this included 47 payers and 5.68 million unique individuals. While approximately 50% (about 1.2 million per year) of self-insured individuals were included in the data, ERISA-based self-insured are not required to submit their claims and approximately 75% of these individuals are not in the data. CIVHC matched the individual identifiers from UCHealth's EHR to the APCD, and had an 80% match rate (i.e., 80% of the UCHealth sample had claims in the APCD). From CIVHC we obtained the medical claims, pharmaceutical claims, and insurance enrollment data across all payers for the matched individuals, and a scrambled identifier that could be used to link the APCD data to the medication order data. This UCHealth EHR – APCD linked dataset was our study data (**eFigure 1**).

Sample Selection. We excluded all orders for patients who had interruptions in pharmacy coverage in the APCD for 12 months before the order (24.9%) and 3 months after the order (6.0%). We required this continuous enrollment to measure whether patients had a prior diagnosis of diabetes, whether the GLP-1RA order was a new initiation of the medication, and whether the order was filled by the patient within 90 days. We excluded orders where patient characteristics (ZIP code, race and ethnicity, payer) were missing (2.7% of orders) or when no BMI measurement was recorded within 12 months of the order (2.5%). Finally, we excluded duplicate orders, which were orders placed after another order for the same GLP-1RA medication (i.e., semaglutide) on the same day (5.5%) (**eFigure 1**). If a prescriber ordered two different medications on the same day, these would be considered two orders. Orders for the same GLP-1RA medication placed one or more days apart for the same patient were considered distinct orders.

© 2025 Sarpatwari A et al. *JAMA Health Form*.

Outcomes. Using medication order data linked to pharmacy claims, we constructed the primary outcome, which was a binary indicator of whether there was a pharmacy claim for the GLP-1RA medication within 90 days of the date of the order. For these medication fills, we constructed our secondary outcome, which was the patient's actual paid out-of-pocket cost for the medication. We constructed out-of-pocket cost as the sum of spending in the deductible, coinsurance, and copayment fields on the pharmacy claim, and used the days supply field to standardize the patient out-of-pocket spending to a 30-day supply. Spending was adjusted to 2023 dollars using the seasonally adjusted monthly Consumer Price Index for medical care for all urban consumers.[3]

Covariates. For each medication order, using data from the EHR we created indicators of patient age group (younger than 50, 50-59, 60-69, 70 or older), sex, and insurance type (Traditional Medicare, Medicare Advantage, or commercial). Race and ethnicity were reported directly in the EHR as two separate variables. Following federal guidelines [i], patients of Hispanic or Latino ethnicity were grouped as Hispanic regardless of race. The remaining non-Hispanic patients were categorized by race as follows: non-Hispanic Asian (Asian), non-Hispanic Black (Black), and non-Hispanic White (White). The remaining 1.9% of orders were for patients who were one of 9 other races, were multi-race or mixed race, or where the patient declined to provide race. We excluded these patients because small sample size within race category. We used data on patient body mass index (BMI) from the EHR to classify patients with obesity, defined as BMI >=30. For the majority of the sample, 7,818 (79.4%), we used BMI measured on the same date as the GLP-1RA medication order. When BMI data from the same date was not available, we used BMI measured for that patient during the most recent encounter in the preceding year (n=2,030, 20.6% of sample). We measured whether a patient had a diagnosis of

© 2025 Sarpatwari A et al. *JAMA Health Form*.

Diabetes Type 2 in the prior year (ICD10-CM containing "E11.x") recorded in either the UCHealth EHR data or the APCD data, or if the patient had a prior order for insulin, metformin, sodium glucose cotransportor-2 inhibitor, or dipeptidyl peptidase-4 inhibitor in the prior year. These patients were classified as having diabetes. We then constructed a categorical variable for indication for the medication order which included diabetes only, obesity only, diabetes and obesity, and neither. We measured whether a medication order was a new initiation of the GLP-1RA, defined as an order for an individual with no other orders or claims for a GLP-1RA in the six months prior to the date of the order. Sixty-two percent of orders (n=6,094) were new initiations and all other orders were classified as subsequent orders.

We used national 2019 American Community Survey 5-year estimates of ZIP code-level median income to calculate income quartiles for patient geocoded census block,[4] available in the All-Payer Claims Database, and assigned each patient to an income quartile based on their ZIP code. We used Rural-Urban Commuting Area (RUCA) codes to identify whether patients lived in rural areas; ZIP codes with RUCA codes 1-7 were classified as urban and all others were classified as rural.

Analysis Methods

We used multivariable logistic regression including the covariates described above to evaluate the association between a patient filling an order for a GLP-1RA, patient race/ethnicity, and indication. Results are reported as predicted probabilities based on this model. For the models of the outcome out-of-pocket cost per 30d prescription, we used multivariable generalized linear models specifying a gamma distribution with a log link function and included the same set of covariates to evaluate the association between patient 30-day out-of-pocket cost

© 2025 Sarpatwari A et al. *JAMA Health Form*.

for their GLP-1RA, patient race/ethnicity, and indication. We selected the gamma distribution because the distribution of out-of-pocket cost in the data was right skewed. All models included month and year fixed effects to control for secular trends, and robust standard errors clustered at the patient level to account for intra-patient correlation across orders. Analyses were conducted using Stata, version 18 in 2024, and we used the margins command to report results as predicted probabilities and predicted values based on coefficients from the regression models. This project was approved by the Colorado Multiple and the Harvard Longwood Campus Institutional Review Boards.

© 2025 Sarpatwari A et al. *JAMA Health Form*.

References

1. Goodman RA, Posner SF, Huang ES, Parekh AK, Koh HK. Defining and Measuring Chronic Conditions: Imperatives for Research, Policy, Program, and Practice. *Prev Chronic Dis*. 2013;10:120239. doi:10.5888/pcd10.120239

2. Salive ME. Multimorbidity in Older Adults. *Epidemiologic Reviews*. 2013;35(1):75-83. doi:10.1093/epirev/mxs009

3. U.S. Bureau of Labor Statistics. Consumer Price Index for All Urban Consumers: Medical Care in U.S. City Average [CPIMEDSL]. FRED, Federal Reserve Bank of St. Louis. Accessed February 20, 2020. https://fred.stlouisfed.org/series/CPIMEDSL

4. US Census Bureau. American Community Survey (ACS). Published online 2019. www.census.gov/programs-surveys/acs

© 2025 Sarpatwari A et al. *JAMA Health Form*.

eFigure 1. Study Sample Selection

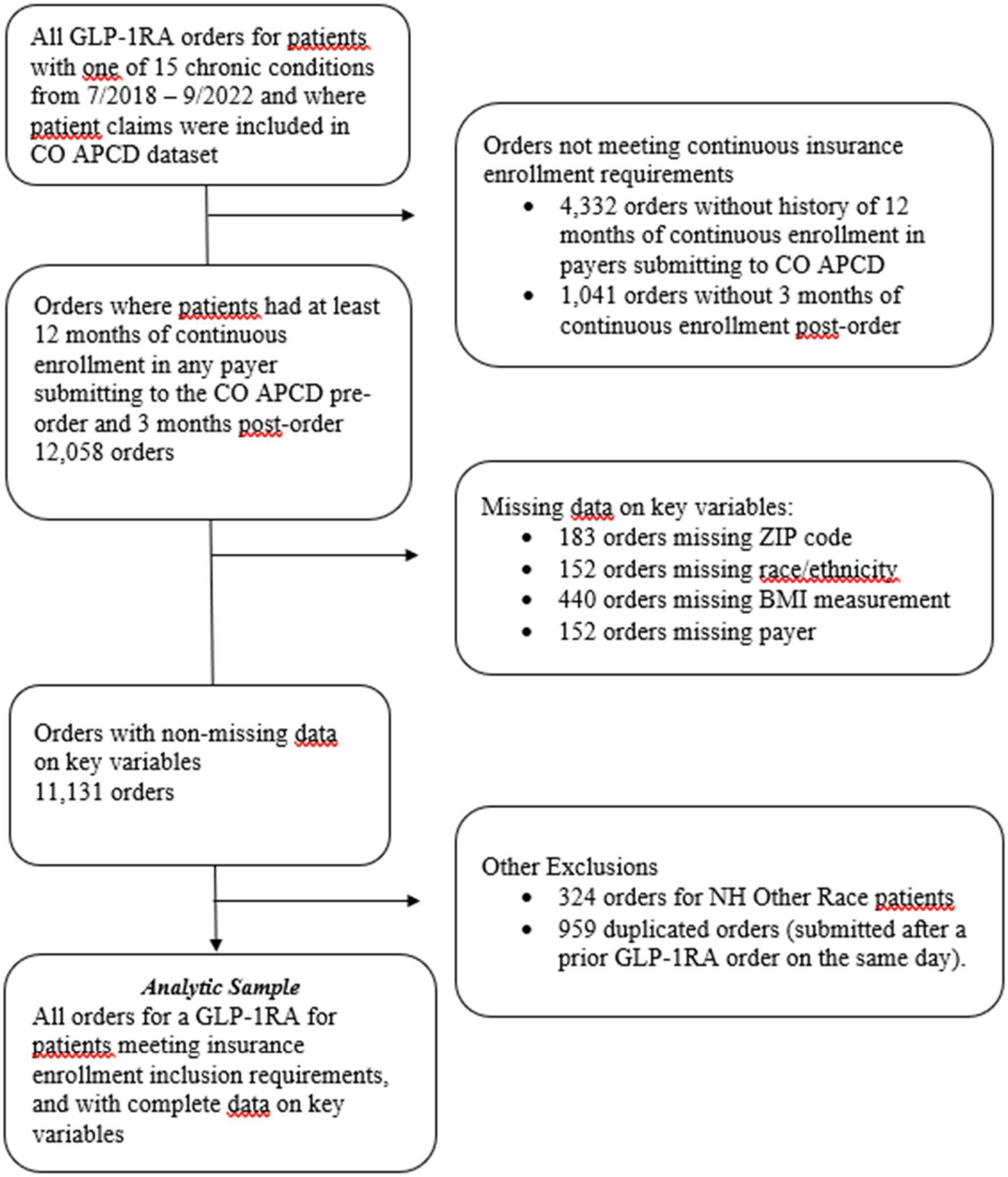

Abbreviations: GLP-1RA, glucagon-like peptide-1 receptor agonist; CO APCD, Colorado All-Payer Claims Database; UCHealth, University of Colorado Health System; BMI, Body Mass Index; NH, Non-Hispanic

© 2025 Sarpatwari A et al. *JAMA Health Form*.

eTable 1. Chronic Conditions Included in Sample	
**Condition**	**Diagnosis Codes**
Arthritis	E83.59, G54.9, M05.0-M06.9, M08.061-62, M08.72, M08.1, M08.419, M08.431, M10.9-M11.9, M12.10-19, M13.0, M14.80, M15.0-M1A.9XX1, M22.40-2, M23.203-4, M23.300-2, M23.306-9, M23.341-59, M23.8X1-9, M24.131-2, M24.28, M24.7, M25.331-9, M25.78-80, M25.841-9, M26.69, M30.3, M35.2, M43.05, M45.0-9, M46.1, M47.1-9, M48.3, M48.8X*, M50.00, M50.3-8, M51.06, M51.34-7, M51.84, M51.6, M51.9, M53.1, M53.9, M62.89, M65.80, M65.839, M70.40-2, M71.461-2, M75.00-2, M76.40-2, M77.20-22, M77.8-9, M89.49, M91.0, M94.1-29, S69.81XA-2XS, T84.060A-3S
Asthma	B44.81, J06.9, J20.9, J33.9, J44.1, J44.9-45.998, J66.8, J67.8, J68.3, J68.8, J69.8, R05, T39.015A, Z57.8-9, Z79.2-4, Z88.6
Atrial Fibrillation	D68.69, I48.0-21, I48.91-2
Cancer	A63.0, B10.89, B27.09, C*, D01.*-D05.*, D07.*-D09.*, D13.4, D21.9, D27.9, D37.6, D38.1, D39.*, D42.9, D43.2, D45-D47.*, D48.9, D49.*, D69.6, D70.9, D72.11*, D72.828, D72.89, D75.0, D75.8*, D76.3, I78.0, L92.8, M07.641, M31.2, O9A111-12, Q78.3, Q82.8, Q87.89, Q93.89, R68.89, R85.614, Z08, Z15.*, Z19.*, Z51.89, Z85.118, Z85.43-4
Chronic Obstructive Lung Disease	E88.01, G47.36, J43.1-44.0, J68.4, J84.10, J96.00, J98.19-4, P25.0, Q32.4, R06.03, R09.02
Chronic Renal Failure	E79.0, E83.52, E87.3, I13.11, I27.20, I27.29, M32.14, N03.9, N17-19, P96.0, Z53.20, Z84.1, Z99.2
Congestive Heart Failure	G47.37, I09.81, I25.5, I38, I42.9-43, I50.1-9, I51.7, I97.130-31, I97.3, P29.0, Z01.810
Coronary Artery Disease	A52.03, A52.06, I20.0-1, I20.9-21.02, I21.3, I21.9, I21.A9, I24.0, I24.9-25.2, I25.41-42, I25.700-84, I25.89-9, I51.5, O99.411-3, Q24.5-8, Q25.49, Q25.79, R93.1, S25.819A, T81.718A, T82.213A-8S, T82.518A, T82.857A-S, T82.867A-S, T82.898A, T82.9XXA-S, Z03.89, Z95.5, Z98.61,

© 2025 Sarpatwari A et al. *JAMA Health Form*.

Dementia	A50.45, A52.17, A81.00, A81.01, A81.2, A81.9, A86, B00.4, B69.0, B99.9, E51.9, E52, E53.8, E56.0, E61.1, E61.9, E85.4, F01.*-F03.*, F05, F06.7*, F07.81, F10.27, F10.97, F13.27, F13.97, F13.988, F18.17, F18.27, F18.97, F19.17, F19.27, F19.97, F20.5, F39, F84.2, G10, G12.21, G20, G23.1, G30.0-31.09, G31.83-85, G31.89-9, G91.2, G91.9, G93.1, I62.03, I67.6, I67.850-58, I68.0, Q90.9, R25.2, R41.0, R41.81, R45.89, S06.9X0A-AS, T57.2X1A, T58.91XA, W88.8XXA, Z77.018, Z91.83
Depression	B94.8, F06.1, F06.31, F09, F10.129, F10.139, F10.14, F10.229, F10.239-24, F10.929, F10.939-94, F11.129-14, F11.229-24, F11.929-94, F13.129, F13.139-14, F13.229, F13.239-24, F13.929, F13.939-94, F14.129-14, F14.229-24, F14.929-94, F15.94, F16.129-14, F16.229-24, F16.288, F16.929-94, F16.988, F18.129-14, F18.229-24, F18.288, F18.929-94, F19.929, F19.939-94, F25.1, F31.0, F31.3-5, F31.70, F31.72, F31.75-75, F31.81, F31.9-F33.9, F34.1, F41.8-9, F43.20-21, F43.23, F45.0, F48.8, F51.05, F53.0, F91.8, G44.89, O99.340-5, R45.851, S09.90XA, T43.625A, T81.89XD-S
Diabetes	B35.1, E03.1, E03.9, E08-E13, E23.2, E27.1, E31.8, E34.328-9, E43, E46, E66.01, E72.09, E72.89, E74.00, E74.09, E75.5, E78.9, E83.110-8, E84.8-9, E87.0, E88.1, E89.1, F70, G11.9, G47.419, G52.9-53, G54.1-2, G54.8, G56.00, G56.20, G57.90, G58.7, G63, G71.3, G98.8-99.2, H16.8, H21.1X9, H33.40, H34.9, H35.049, H35.059, H35.09, H35.52, H35.81-82, H40.009, H40.89. H42, H43.10-13, H43.811-19, H7.099, H47.20, H49.00, H49.40, H51.20, H54.0X33, H54.0X44, H54.0X55, H54.10, H54.2X11, H54.2X22-40, H54.50, H54.60, H54.8, H57.9, H90.5, H91.9, I70.0, I89.0, K00.4, K06.9, K31.84, K31.9, K52.9, K63.9, K74.1, K86.89-9, K92.9, L03.039, L03.119, L08.9, L14, L54, L68.0, L81.8, L83-4, L89.509, L89.600-604, L89.609, L89.890-94, L89.899, L89.91, L97.101-4, L97.109-14, L97.1-L98.9, M14.849, M14.879, M21.969, M54.10-17,

© 2025 Sarpatwari A et al. *JAMA Health Form*.

	M62.20, M62.50, M62.81, M79.89, M85.2, M86.9, M90.80, N05.8, N08, N28.9, N52.1, N52.9, N64.89, O24.011-25.13, O99.810, P05.9, P70.1-2, P78.3, P90, Q02, Q04.3, Q04.9, Q10.3, Q45.0, Q55.8, Q68.8, Q82.4, Q87.11, Q99.8, R16.0, R19.7, R23.8, R45.4, R62.50, R62.52, R73.03-9, S36.2095, S42.411B, S43.102S, S92.901A-9S, T38.0X1A-5S, T39.395A, Z79.2-4, Z79.84-9, Z86.31-9, Z89.619, Z90.410, Z96.41, Z98.890
Hyperlipidemia	E74.89, E78.0-6, R79.89, T86.99,
Hypertension	D35.00, D53.1, E25.0, E26.09, E27.40, E27.9, E34.9, E72.59, E87.6, E87.79, E88.09, G93.2, I10-13.10, I13.2-16.9, I61.5, I62.9, I67.4, I67.83, I70.1, I97.88, J81.1, N25.9, N26.2, N28.89, O10.011-03, O10.211-23, O10.32-11.9, O13.1-14.95, O15.1-16.9, O99.280, O99.43, P29.2, P52.5, P52.8, Q11.2, Q61.3, Q73.8, R00.0, R00.9, R03.0, T38.4X1A, T45.1X1A, T50.901A-4S, T80.89XD-S, Z90.5, Z94.9
Osteoporosis	E70.29, M80.00XA-81.6, M84.459A-S, M85.89, M87.239-43, M89.8X9, Q25.1
Stroke	D57.1, D68.59, D72.820, E88.81, F06.30, F07.0, G12.22, G21.8, G43.909, G45.0-1, G45.3, G45.8-46.4, G51.0, G81.90, G83.10, G83.20, G83.9, G97.81-82, H53.469, H53.9, I60.7, I60.9, I61.9, I63.*, I69.30, I69.319-22, I69.339, I69.349, I69.359, I69.369, I69.391-92, I69.398, I70.90, I74.10, I74.9, I77.70, I97.810-11, I97.89, P52.3, P91.821-2, P91.829, P96.89, R20.9, R26.9, R27.9, R29.810, R40.20, R41.89, R44.8, R47.1, R51.9, R53.1, S06.6X1A-9S, Z86.73

Notes: Arthritis includes ankylosing spondylitis, osteoarthritis, and rheumatoid arthritis; Stroke includes transient ischemic attack.

© 2025 Sarpatwari A et al. *JAMA Health Form*.

eTable 2. GLP-1RA Medications Included in Study

Generic Names
Albiglutide
Dulaglutide
Exenatide
Liraglutide
Lixisenatide
Semaglutide
Tirzepatide
Brand Names
Adlyxin
Byetta
Bydureon
Mounjaro
Rybelsus
Saxenda
Tanzeum
Trulicity
Victoza
Wegovy[1]

Notes: Abbreviations: GLP-1RA, glucagon-like peptide-1 receptor agonist
1. Wegovy was approved by the FDA in June 2021, and thus only available in the latter part of the study period.

---

[i] https://orwh.od.nih.gov/toolkit/other-relevant-federal-policies/OMB-standards

© 2025 Sarpatwari A et al. *JAMA Health Form*.
