# Supplementary material for: Glucagon-Like Peptide-1 Receptor Agonist Order Fills and Out-of-Pocket Costs by Race, Ethnicity, and Indication
Source: JAMA Health Forum. 2025 Oct 10;6(10):e254258. doi: 10.1001/jamahealthforum.2025.4258 (PMC12514621; doi:10.1001/jamahealthforum.2025.4258)
Supplement: Supplement 2. — Data Sharing Statement [file jamahealthforum-e254258-s002.pdf]

Data Sharing Statement

Sarpatwari. Glucagon-Like Peptide-1 Receptor Agonist Order Fills and Out-of-Pocket Costs by Race, Ethnicity, and Indication. *JAMA Health Forum*. Published October 10, 2025. doi:10.1001/jamahealthforum.2025.4258

Data
Data available: No

Additional Information
Explanation for why data not available: The data we are using come from two proprietary sources, which do not permit data sharing.
